# Larvicidal activity and structure activity relationship of cinnamoyl amides from Zanthoxylum armatum and their synthetic analogues against diamondback moth, Plutella xylostella 

**DOI:** 10.17179/excli2016-166

**Published:** 2016-03-18

**Authors:** Vishal Kumar, S. G. Eswara Reddy, Anuja Bhardwaj, Shudh Kirti Dolma, Neeraj Kumar

**Affiliations:** 1 Natural Product Chemistry and Process Development Division, CSIR-Institute of Himalayan Bioresource Technology, Palampur-176061, Himachal Pradesh, India; 2 Entomology Laboratory, CSIR-Institute of Himalayan Bioresource Technology, Palampur-176061, Himachal Pradesh, India

**Keywords:** cinnamoyl amides, structure activity relationship, larvicidal activity, Plutella xylostella

## Abstract

Cinnamoyl amides isolated from *Zanthoxylum* *armatum* (Rutaceae) and their synthetic analogues were tested for their insecticidal activity against the second instar larvae of diamondback moth, *Plutella xylostella* (L.) (Lepidoptera: Yponomeutidae) to determine the promising structures with insecticidal activity. Most of the test compounds showed promising activity against larvae of* P. xylostella*. However, the activities of different compounds varied depending on the presence of different substituents at various positions of both the aromatic rings A and B. Among the tested compounds, **8**,* N*-(3-bromo-4-methoxyphenethyl)cinnamamide showed best larvicidal activity with an LC_50_ = 62.13 mg/L followed by **6**, *N*-(3׳-bromophenethyl)cinnamamide (LC_50_=128.49 mg/L) and **2*** N*-(4׳-methoxyphenylethyl)cinnamamide (LC_50_ = 225.65 mg/L).

## Introduction

The diamondback moth, *Plutella xylostella* (L.) (Lepidoptera: Yponomeutidae) is most damaging insect pest of cruciferous crops throughout the world (Talekar, 1992[22]) and greatest threat to crucifer production in many parts of the world, causing more than 90 % crop loss (Harcourt, 1962[10]; Talekar and Shelton, 1993[23]; Verkerk and Wright, 1996[24]; Gu et al., 2010[9]). Intensive use of chemical pesticides in its control has led to this pest developing resistance to a wide range of insecticides and caused serious damage to natural enemies (Harcourt, 1962[10]; Ke et al., 1991[11]). Several synthetic insecticides besides botanical and microbial control agents have been used for the control of this pest (Liu et al., 1982[15]; Srinivasan and Kumar, 1982[20]; Chaudhuri et al., 2001[4]). Nearly two decades ago, the annual cost of controlling *P. xylostella *on a worldwide basis was estimated to be US $ 1 billion but in a recent study the overall management costs were estimated at US$ 4 billion (Zalucki et al., 2012[27]).

Throughout the world, pesticides have dominated attempts to control *P. xylostella* for more than 40 years (Talekar and Shelton, 1993[23]; Syed, 1992[21]).The negative impacts of pesticides and increasing pesticide resistance have increased the interest in alternative control methods, with emphasis being placed on biological control, host plant resistance, cultural control, botanicals and other non-polluting methods (Cheng, 1988[5]; Lim et al., 1996[14]). Due to harmful effects of synthetic pesticides to health, environment and resistance development in pests, there is a need for the development of safer and effective alternate strategies to contain the pests. 

*Zanthoxylum armatum* DC. (Rutaceae) is found abundantly throughout the Western Himalayas at altitudes of 1200-3000 m and is extensively used in the Indian system of medicine, as carminative, stomachic and anthelmintic. The extracts of this plant are known to possess insecticidal, anti-fungal and anti-microbial activities (Singh and Singh, 2011[19]). Various pharmacological activities of this plant are attributed to the presence of amides as cinnamoyl amides isolated from various *Zanthoxylum* species and other plants have shown a wide spectrum of biological activities such as antiinflammatory, antiplasmodial, antiviral, antibacterial, antiplatelet aggregation, eukotriene biosynthesis in human polymorphonuclear leukocytes and anticancer activities (Wu et al., 1995[26]; Ross et al., 2004[18]).

Insecticidal activity of several amides has been reported against Lepidopetran insects (Ewete et al., 2000[8]; Park et al., 2002[16]; Dyer et al., 2003[6]; Batista-Pereira et al., 2006[2]). However, cinnamoyl amide conjugates of phenylethylamine derivatives isolated from *Z. armatum* or any other plant have not been investigated previously for their insecticidal activities. Keeping in view the great potential of cinnamoyl amides of plant origin, present study was conducted to screen a series of cinnamoyl amides isolated from *Z. armatum* and their synthetic analogues for their larvicidal activity against *P. xylostella* and determine structure activity relationship (SAR).

## Materials and Methods

### General

Boric acid used for the synthesis of silica-supported boric acid (H_3_BO_3_-SiO_2_) was purchased from Ranbaxy Chemicals Ltd. Silica gel (60-120 mesh) used for preparation of H_3_BO_3_-SiO_2_ catalyst and column chromatography, was purchased from Sisco Research Laboratories Pvt. Ltd., India. The course of the reactions was monitored by TLC on pre-coated aluminium plates (silica gel 60 F_254_) purchased from Merck, Germany. All other chemicals were purchased from Sigma-Aldrich, USA and were used without further purification. NMR spectra were recorded on Bruker Avance-300 and 600 spectrometers at room temperature using CDCl_3_ or DMSO as solvents and TMS as internal standard.

### Extraction and isolation of compounds (10, 12 and 13) from Z. armatum

Air dried powder of bark (1.0 Kg) of *Z. armatum* was extracted with methanol: H_2_O (80: 20; v/v, 3 × 4L) in a percolator at room temperature for 12 h. Combined percolations were dried under reduced pressure to yield 238.2 g of crude extract. The extract thus obtained was suspended in water and sequentially fractionated with *n*-hexane, chloroform, ethyl acetate and *n*-butanol, and dried under vacuo to get corresponding fractions i.e. *n*-hexane (12.5 g), chloroform (34.3 g), ethyl acetate (12.1 g), *n*-butanol (92.4 g) and aqueous fraction (78.1 g). Chloroform fraction (25.0 g) was subjected to column chromatography over silica-gel (60-120 mesh) and eluted with 10, 20, 30, 50, 75 and 100 % ethyl acetate in *n*-hexane (5 x 200 mL each). Repeated column chromatography of fractions obtained in 50 % ethyl acetate/*n*-hexane led to the isolation of armatamide (**12**, 480 mg). Chromatographic purification of fractions eluted in 75 % ethyl acetate/*n*-hexane resulted in the isolation of zanthosin (**10**, 57 mg) and rubimamin (**13**, 23 mg).

### Synthesis of silica-supported boric acid (H_3_BO_3_-SiO_2_)

H_3_BO_3_-SiO_2_ was synthesized by following our previously reported procedure (Kumar et al., 2011[13]).

### General experimental procedure for the synthesis of cinnamoyl amides 

To a stirred mixture of phenethylamine derivative (1 mmol) and cinnamoyl chloride derivative (1.1 mmol) in toluene (4 mL) at room temperature, silica-supported boric acid (H_3_BO_3_-SiO_2_, 1.5 mol %) was added. The reaction was kept at room temperature and progress of the reaction was monitored by TLC. After completion of the reaction, ethyl acetate (5 mL) was added and the catalyst was separated by ﬁltration. The filtrate thus obtained was washed with brine (3×5 mL) and dried over anhydrous sodium sulphate. The product was purified by crystallization with ethanol. Isolated compounds were characterized by ^1^H and ^13^C NMR spectroscopy.

***N*-(Phenylethyl)cinnamamide (1) **^1^H NMR (300 MHz, CD_3_OD) *δ*_H_ 2.84-2.89 (m, 2H), 3.51-3.56 (m, 2H), 6.60 (d, 1H, *J *= 15.8 Hz), 7.20-7.23 (m, 1H), 7.26-7.29 (m, 4H), 7.36-7.41 (m, 3H), 7.51-7.56 (m, 3H); ^13^C NMR (75 MHz, CD_3_OD) *δ*_C_ 35.5, 41.2, 120.8, 126.3, 127.8, 128.5, 128.8, 128.9, 129.7, 135.2, 139.5, 140.6, 167.6. 

***N*-(4**׳**-Methoxyphenylethyl)cinnamamide (2) **^1^H NMR (300 MHz, CD_3_OD) *δ*_H_ 2.88-2.93 (m, 2H), 3.47-3.52 (m, 2H), 3.79 (s, 3H), 6.61 (d, 1H, *J* = 15.7 Hz), 6.84-6.92 (m, 2H), 7.14-7.21 (m, 3H), 7.37-7.41 (m, 2H), 7.50-7.51 (m, 3H); ^13^C NMR (75 MHz, CD_3_OD) *δ*_C_ 33.7, 39.6, 55.2, 114.4, 120.9, 127.8, 128.6, 128.9, 129.7, 129.8, 135.3, 140.6, 159.3, 167.6. 

***N*-(2**׳**-Methoxyphenylethyl)cinnamamide (3) **^1^H NMR (300 MHz, CD_3_OD) *δ*_H_ 2.86-2.90, 3.49-3.53 (m, 2H), 3.83 (s, 3H), 6.59 (d, 1H, *J* = 15.7 Hz), 6.87-6.95 (m, 3H), 7.14-7.20 (m, 2H), 7.36-7.38 (m, 3H), 7.53-7.56 (m, 2H); ^13^C NMR (75 MHz, CD_3_OD) *δ*_C_ 30.3, 39.8, 54.7, 110.4, 120.5, 120.9, 127.4, 127.8, 127.9, 128.9, 129.7, 130.4, 135.3, 140.5, 158.1, 167.6. 

***N*-(3**׳**,4**׳**-Dimethoxyphenethyl)cinnamamide (4) **^1^H NMR (300 MHz, CD_3_OD) *δ*_H_ 2.89-2.94 (m, 2H), 3.50-3.55 (m, 2H), 3.82 (s, 3H), 3.84 (s, 3H), 6.61 (d, 1H, *J* = 15.9 Hz), 6.78-6.94 (m, 3H), 7.37-7.39 (m, 3H), 7.50-7.56 (m, 3H); ^13^C NMR (75 MHz, CD_3_OD) *δ*_C_ 33.1, 41.0, 55.5 (2 OCH_3_), 112.4, 112.8, 121.2, 127.8, 128.3, 128.9, 129.8, 132.4, 135.2, 140.6, 148.8, 149.8, 167.6.

***N*-(2**׳**-Bromophenethyl)cinnamamide (5) **^1^H NMR (300 MHz, CD_3_OD) *δ*_H_ 3.01-3.06 (m, 2H), 3.54-3.59 (m, 2H), 6.60 (d, 1H, *J* = 15.6 Hz), 7.13-7.24 (m, 2H), 7.29-7.32 (m, 2H), 7.36-7.40 (m, 3H), 7.50-7.61 (m, 3H); ^13^C NMR (75 MHz, CD_3_OD) *δ*_C_ 33.9, 39.5, 120.8, 124.2, 128.3, 128.9, 129.3, 129.8, 131.1, 132.9, 133.3, 135.2, 138.7, 140.7, 167.7.

***N*-(3**׳**-Bromophenethyl)cinnamamide (6) **^1^H NMR (300 MHz, CD_3_OD) *δ*_H_ 2.98-3.02 (m, 2H), 3.56-3.61 (m, 2H), 6.58 (d, 1H, *J* = 15.5 Hz), 7.17-7.26 (m, 3H), 7.36-7.45 (m, 3H), 7.52-7.58 (m, 4H); ^13^C NMR (75 MHz, CD_3_OD) *δ*_C_ 34.0, 39.3, 121.3, 125.4, 127.1, 128.5, 128.9, 129.3, 130.9, 133.0, 133.8, 135.2, 137.9, 140.1, 167.5.

***N*-(4**׳**-Bromophenethyl)cinnamamide (7) **^1^H NMR (300 MHz, CD_3_OD) *δ*_H_ 2.96-3.01 (m, 2H), 3.55-3.59 (m, 2H), 6.63 (d, 1H, *J* = 15.9 Hz), 7.21-7.28 (m, 3H), 7.41-7.50 (m, 3H), 7.54-7.59 (m, 4H); ^13^C NMR (75 MHz, CD_3_OD) *δ*_C_ 33.4, 39.0, 119.2, 126.2, 127.4, 128.2, 129.3, 130.0, 130.5, 134.6, 137.3, 139.2, 167.2.

***N*-(3**׳**-Bromo-4**׳**-methoxyphenethyl)cinnamamide (8) **^1^H NMR (300 MHz, CD_3_OD) *δ*_H_ 2.84-2.89 (m, 2H), 3.46-3.52 (m, 2H), 3.90 (s, 3H), 6.63 (d, 1H, *J* = 15.7 Hz), 6.75 (d, 1H, *J* = 7.9 Hz), 7.13-7.19 (m, 2H), 7.28-7.36 (m, 3H), 7.46-7.53 (m, 3H); ^13^C NMR (75 MHz, CD_3_OD) *δ*_C_ 33.8, 39.7, 55.6, 108.4, 113.4, 120.1, 127.9, 128.2, 128.9, 129.3, 129.7, 130.2, 132.0, 136.5, 141.3, 167.5.

***N*-(2**׳**-Fluorophenethyl)cinnamamide (9) **^1^H NMR (300 MHz, CD_3_OD) *δ*_H_ 3.05-3.11 (m, 2H), 3.54-3.60 (m, 2H), 6.56 (d, 1H, *J* = 15.3 Hz), 7.01-7.07 (m, 1H), 7.18-7.29 (m, 3H), 7.35-7.46 (m, 3H), 7.56-7.64 (m, 3H); ^13^C NMR (75 MHz, CD_3_OD) *δ*_C_ 34.7, 40.5, 121.8, 121.9, 124.4, 128.0, 128.4, 129.1, 129.7, 131.5, 131.7, 132.6, 132.7, 135.7, 138.3, 141.8, 143.2, 167.9.

**Rubimamin (10)** ^1^H NMR (300 MHz, CDCl_3_) *δ*_H_ 2.83 (t, 2H, *J* = 6.9 Hz), 3.62-3.66 (m, 2H), 3.83-3.93 (m, 12H), 6.24 (d, 1H, *J* = 15.5 Hz), 6.74-6.84 (m, 4H), 6.99 (d, 1H, *J* = 1.8 Hz), 7.05 (dd, 1H, *J* = 1.8 Hz, 8.1 Hz), 7.56 (d, 1H, *J* = 15.5 Hz); ^13^C NMR (75 MHz, CDCl_3_) *δ*_C_ 35.6, 41.3, 56.3 (4 OCH_3_), 110.1, 111.5, 111.8, 112.4, 118.9, 121.0, 122.3, 128.1, 131.8, 141.2, 148.1 149.4, 149.5, 150.9, 166.6; HR-ESI-MS calcd. for C_21_H_26_NO_5 _[M + H]^+ ^*m/z* 372.1811, found 372.1802.

***N*-(Phenylethyl)-3,4-methylenedioxycinnamamide (11) **^1^H NMR (300 MHz, CD_3_OD) *δ*_H_ 2.83-2.88 (m, 2H), 3.49-3.54 (m, 2H), 5.97 (s, 2H), 6.40 (d, 1H, *J* = 15.6 Hz), 6.82 (d, 1H, *J* = 8.0 Hz), 7.01 (dd, 1H, *J* = 1.5 Hz, 8.0 Hz), 7.07 (d, 1H, *J* = 1.5 Hz), 7.19-7.29 (m, 5H), 7.44 (d, 1H, *J* = 15.6 Hz); ^13^C NMR (75 MHz, CD_3_OD) *δ*_C_ 35.6, 41.2, 101.8, 106.0, 108.3, 118.7, 124.0, 126.3, 128.5, 128.8, 129.6, 139.5, 140.5, 148.8, 149.6, 167.8. 

**Armatamide (12) **^1^H NMR (300 MHz, DMSO-d_6_) *δ*_H_ 2.70 (t, 2H, J = 6.9 Hz), 3.72 (s, 3H), 3.73-3.78 (m, 2H), 6.06 (s, 2H), 6.45 (d, 1H, *J* = 15.5 Hz), 6.88 (d, 2H, *J* = 7.0 Hz), 6.94 (d, 1H, *J* = 8.0 Hz), 7.05 (d, 1H, *J* = 8.0 Hz), 7.13-7.15 (m, 3H), 7.33 (d, 1H, *J* = 15.5 Hz); ^13^C NMR (75 MHz, DMSO-d_6_) *δ*_C_ 33.7, 39.9, 54.3, 100.8, 105.6, 107.9, 113.1, 119.7, 122.5, 128.7, 128.9, 130.7, 137.7, 147.3, 147.8, 157.1, 164.4; HR-ESI-MS calcd. for C_19_H_20_NO_4 _[M + H]^+ ^*m/z* 326.1392, found 326.1377.

**Zanthosin (13) **^1^H NMR (300 MHz, CDCl_3_) *δ*_H_ 2.84 (t, 2H, J = 6.9 Hz), 3.61-3.65 (m, 2H), 3.86 (s, 6H), 5.98 (s, 2H), 6.18 (d, 1H, *J* = 15.5 Hz), 6.74-6.88 (m, 4H), 6.95-7.00 (m, 2H), 7.53 (d, 1H, *J* = 15.5 Hz); ^13^C NMR (75 MHz, CDCl_3_) *δ*_C_ 35.6, 41.3, 56.2, 56.3, 101.8, 106.7, 108.8, 111.8, 112.4, 119.1, 121.0, 124.1, 129.6, 131.8, 141.1,148.1, 148.6, 149.4, 149.5, 166.4; HR-ESI-MS calcd. for C_20_H_22_NO_5 _[M + H]^+ ^*m/z* 356.1498, found 356.1481.

***N*-(1-Hydroxy-1-phenylethyl)cinnamamide (14) **^1^H NMR (300 MHz, CD_3_OD) *δ*_H_ 3.57-3.60 (m, 2H), 4.82-4.89 (m, 1H), 6.67 (d, 1H, *J* = 15.7 Hz), 7.28-7.57 (m, 11H); ^13^C NMR (75 MHz, CD_3_OD) *δ*_C_ 46.4, 70.0, 120.7, 125.9, 126.1, 127.6, 128.3, 128.9, 129.8, 135.2, 140.8, 142.9, 167.9. 

### Residual toxicity of cinnamoyl amides and their analogues against P. xylostella 

#### Test insect

*P. xylostella* used for the experimental study was collected from cabbage (*Brassica oleracea* L.) field and reared on mustard, *Brassica juncea* (L.) seedlings in the laboratory for more than 50 generations at 26 ± 2 °C temperature, 60 ± 5 % relative humidity and photoperiod 16:8 L:D). Second/third instar larvae starved were used for the experiments. 

#### Preliminary screening 

Preliminary screening of cinnamoyl amides and their analogues at higher concentrations (10000 and 5000 ppm) were tested for their toxicity against second instar larvae of *P. xylostella.* Based on preliminary screening results, five concentrations were fixed and tested against target pest in the main experiment.

#### Residual toxicity of cinnamoyl amides and their analogues 

Residual toxicity of ginger extracts and ginger oil against *P. xylostella* was tested following leaf dip bio-assay (Park et al., 2002[16]; Kumar et al., 2016[12]; Reddy et al., 2015[17]) against second instar larvae of *P. xylostella*. Five concentrations (62.5 to 1000 mg/L) of test compounds were prepared either separately or by serial dilution from the solution of higher concentration. Commercial insecticide (chlorpyriphos at 25 to 400 mg/L) commonly used for the control of *P. xylostella* was used as a positive control. Observations on mortality were recorded at 72 and 96 h intervals. 

### Data analysis

Data from all bioassays were corrected for control mortality using Abbott formula (Abbot, 1925[1]) and analyzed using SPSS 7.5 for calculating LC_50_ values (concentration that causing 50 % mortality) by log-probit regression.

## Results

### Insecticidal activity against P. xylostella

**Figure 1:**
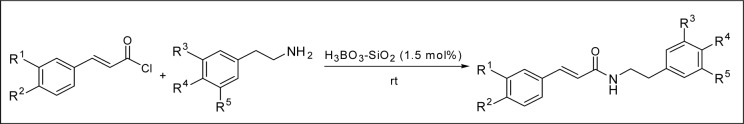
H_3_BO_3_-SiO_2_ catalyzed synthesis of cinnamoyl amides of phenethylamine derivatives

**Figure 2:**
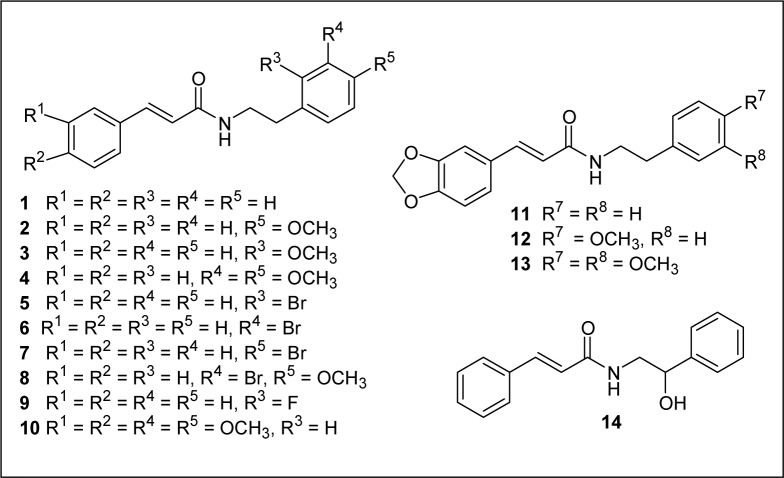
Structures of cinnamoyl amides 1-14

Initially, compounds **10**, **12** and **13** isolated from *Z. armatum* were evaluated for their insecticidal activity against 2^nd^ instar larvae of *P. xylostella*. Compound **12** (armatamide) showed promising activity with an LC_50 _= 298.70 mg L^-1^ after 96 h, whereas compounds **10** and **13** did not show significant activity (Table 1(Tab. 1)). Further, in order to derive structure activity relationship various substituted analogues (**1-9**, **11** and **14**) were synthesized by the reaction of corresponding acid halides and amines in the presence of silica-supported boric acid (H_3_BO_3_-SiO_2_) as catalyst at room temperature (Figure 1(Fig. 1)) and tested for their activity. The structures of tested compounds (**1-14**) are given in Figure 2(Fig. 2) and the results of activities of these compounds against *P. xylostella* in terms of lethal concentration to kill 50 % of the population relative to control (LC_50_ values) and other regression parameters are summarized in Table 1(Tab. 1). 

### Structure activity relationship (SAR)

**Figure 3:**
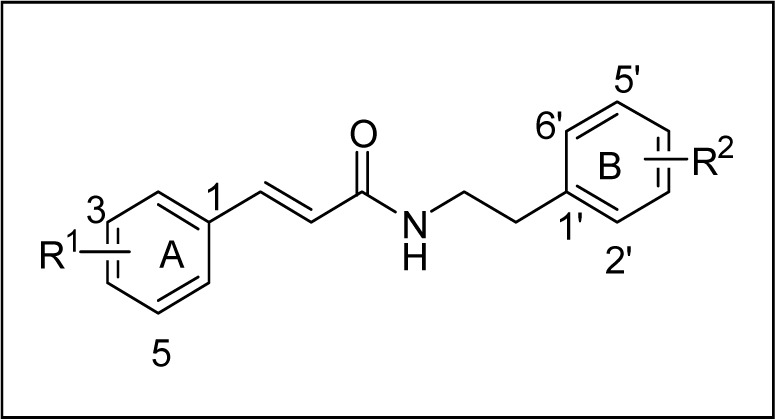
Basic structure of cinnamoyl amides of *Z. armatum*

It is evident that most of the test compounds showed promising activity against larvae of* P. xylostella*. However, the activities of different compounds varied depending on the presence of different substituents at various positions of both the aromatic rings A and B (Figure 3(Fig. 3)). As evident from Table 1(Tab. 1), most of the test compounds exhibited toxicity against *P. xylostella* at 62.5 to1000 mg L^-1^. Compound **8 **showed 96.67 % activity at 1000 mg L^-1^ followed by **2** (86.67 %) and **3** (76.7 %) (Figure 3(Fig. 3)). Probit analysis results showed that, among the tested compounds, **8***, N*-(3-bromo-4-methoxyphenethyl)cinnamamide was most active against larvae of *P. xylostella* with an LC_50_ = 62.13 mg/L, followed by **6**, *N*-(3׳-bromophenethyl)cinnamamide (LC_50 _= 128.49 mg/L) and **2**,* N*-(4׳-methoxyphenylethyl)cinnamamide (LC_50_ = 225.65 mg/L). The LC_50_ values for the other compounds **1, 3, 7, 11, 12 **and **14** were 485.67, 591.85, 412.94, 623.89, 298.70 and 467.90 mg/L respectively.

The unsubstituted amide **1** showed significant activity with an LC_50_ value of 485.67 mg/L. The presence of oxygenated substituents at 3 and 4 positions of ring A (such as methylenedioxy and dimethoxy) reduced the activity as compared to unsubstituted amide **1** (Table 1(Tab. 1), compounds **10-13**). Compound **11** containing 3,4-methylenedioxy (ring A) was found to be 1.4 times less active (LC_50 _= 623.89 mg/L) than **1**. While the presence of 4׳-OCH_3_ (ring B) led to increase in the activity by 2-fold (**2**, LC_50 _= 225.65 mg/L), the presence of 3׳, 4׳-(OCH_3_)_2_ substitution on ring B led to reduction or complete loss in the activity (**4**, **10** and **13**). Compound **12** having 3,4-methylenedioxy and 4׳-OCH_3_ substituents showed LC_50_ value of 298.70 mg/L. Slight decrease in activity was observed for 2׳-OCH_3_ (ring B) substituted derivative (**3**, LC_50 _= 591.85 mg/L). In case of 3׳-Br substituent on ring B (**6**, LC_50_ = 128.49 mg/L), the activity was increased by 4-fold, whereas in case of 4׳-Br substituted cinnamoyl amide, a slight increase in the activity was observed (**7**, LC_50_ = 412.94 mg/ L). As 3׳-Br and 4׳-OCH_3_ substitution showed increase in the activity individually, a compound having both these groups was synthesized (**8**) and was found to be most potent among all the tested compounds with an LC_50_ of 67.31 mg/L. Halogen substituent at 2׳ position (ring B) such as 2׳-Br and 2׳-F had negative influence on the activity as **5** (2׳-Br) gave a very high LC_50_ value of 1582.35 mg/L and **9** (2׳-F) did not show significant activity. The presence of an -OH substituent on aliphatic chain of phenethylamine unit (**14**, LC_50_ = 467.90 mg/L) did not show a significant effect on the activity as compared to unsubstituted amide **1**.

The results from the present study showed that, the amide **8** was most active (LC_50_ = 62.13 mg/L) against second instar larvae of *P. xylostella* and was comparable with the standard insecticide (chlorpyriphos) at 96 h after treatment followed by compound **6**, (LC_50_ = 128.49 mg/L) and compound **2** (LC_50_ = 225.65 mg/L). 

## Discussion

Cinnamoyl amides isolated from *Z.* *armatum* and their synthetic analogues were tested against the second instar larvae of *P. xylostella* for larvicidal activity*. *It is evident that most of the compounds showed larvicidal activity to larvae of* P. xylostella*. However, the activities of different compounds varied depending on the presence of different substituents at various positions of both the aromatic rings A and B. In the present study, amide **8** was found more effective among tested compounds and was comparable with the positive control followed by compound **6** and **2**. Present results are in conformity with the findings of other researchers who tested the different amides against insect pests. Pyrazole amide derivatives containing hydrazone substructures showed promising activity against *P. xylostella*, *Helicoverpa armigera*, *Laphygma exigua*, *Spodoptera litura* at 5, 10, 200, 20, mg/L, respectively (Wu et al., 2012[25]). In a similar study, the amide N-[3-(3,4-methylenedioxyphenyl)-2-(E)-propenoyl] piperidine (Batista-Pereira et al. 2006[2]) and (E)-1-(1-Piperidinyl)-3-[4-(trifluoromethoxy)phenyl]-2-propen-1-one (Castral et al., 2011[3]) was found effective against larvae of *S. frugiperda* with LD_50_ = 1.07µg/mg and 0.793 μg/mg, respectively. Similar results of larval mortality were also observed for different amides isolated from Piper species against larvae of *S. frugiperda* (Dyer et al., 2003[6]), *S. litura* (Park, et al., 2002[16]), *Ostrinia nubilalis* (Ewete et al., 2000[8]) and *Ascioa monusteorseis* (Estrela et al., 2003[7]).

## Conclusions

Most of the test compounds showed promising activity against larvae of* P. xylostella*. However, the activities of different compounds varied depending on the presence of different substituents at various positions of both the aromatic rings A and B. Among the tested compounds, **8**,* N*-(3-bromo-4-methoxyphenethyl)cinnamamide showed best larvicidal activity followed by **6**, *N*-(3׳-bromophenethyl)cinnamamide and **2*** N*-(4׳-methoxyphenylethyl)cinnamamide. 

## Notes

S. G. Eswara Reddy and Neeraj Kumar (Natural Product Chemistry and Process Development Division, CSIR-Institute of Himalayan Bioresource Technology, Palampur-176061, Himachal Pradesh, India; e-mail: neeraj@ihbt.res.in) equally contributed as corresponding authors.

## Acknowledgements

Authors are grateful to Director, CSIR-Institute of Himalayan Bioresource Technology, Palampur for facilities to carry out the study. Financial assistance from Council of Scientific and Industrial Research, New Delhi under the project BSC-0213 is duly acknowledged. Mr. VK is grateful to UGC for Senior Research Fellowship. IHBT communication number for this article is 3620.

## Declaration of interest

The authors declare that they have no conflict of interest.

## Figures and Tables

**Table 1:**
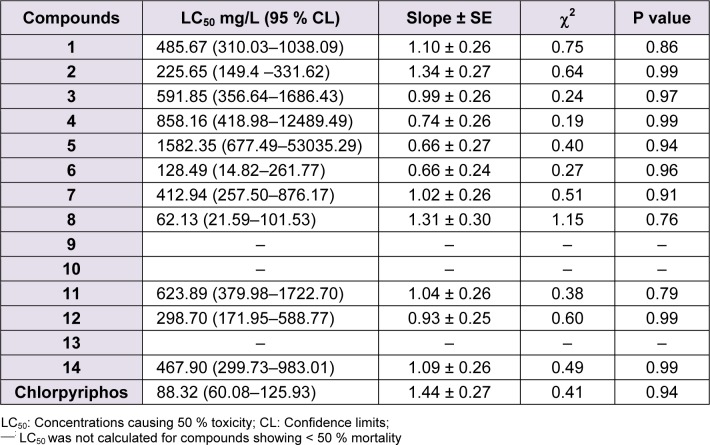
Toxicity of amides against larvae of *Plutella xylostella* after 96 h
